# Group Membership Modulates Fairness Consideration Among Deaf College Students—An Event-Related Potential Study

**DOI:** 10.3389/fpsyg.2022.794892

**Published:** 2022-02-08

**Authors:** Yuqi Gong, Li Yao, Xiaoyi Chen, Qingling Xia, Jun Jiang, Xue Du

**Affiliations:** ^1^Key Laboratory of Applied Psychology, Chongqing Normal University, Chongqing, China; ^2^School of Education, Chongqing Normal University, Chongqing, China; ^3^School of Artificial Intelligence, Chongqing University of Technology, Chongqing, China; ^4^Department of Basic Psychology, School of Psychology, Third Military Medical University, Chongqing, China

**Keywords:** fairness consideration, deaf college students, ultimatum game, ERP, group identity

## Abstract

Group interaction is an essential way of social interaction and plays an important role in our social development. It has been found that when individuals participate in group interactions, the group identity of the interaction partner affects the mental processing and behavioral decision-making of subjects. However, little is known about how deaf college students, who are labeled distinctly different from normal hearing college students, will react when facing proposers from different groups in the ultimatum game (UG) and its time course. In this study, we recruited 29 deaf college students who played the UG in which they received extremely unfair, moderately unfair, or fair offers from either outgroup members (normal hearing college students) or ingroup members (deaf college students), while their brain potentials were recorded. The behavioral results showed that group membership did not impact the acceptance rate of deaf college students. But, event-related potential (ERP) analysis demonstrated an enhanced feedback-related negativity (FRN) elicited by ingroup members compared to outgroup members. Importantly, we found that under fairness conditions, deaf college students induced more positive P2 and P3 facing ingroup members compared to outgroup members. Our results demonstrated that group membership may modulate the performance of deaf college students in the UG and the existence of ingroup bias among deaf college students. This provides some evidence for the fairness characteristics of special populations, so that to improve the educational integration of colleges and universities.

## Introduction

As a code of conduct and ethics in our social interaction, fairness consideration is essential to both the individual survival and social stability (Rawls, 1985). Psychology and behavioral economics often use the ultimatum game (UG) to study fairness consideration. In the UG, according to the hypothesis of “rational assumption,” the proposer should give the responder the least amount of money within the scope permitted by the rules and the responder should accept all the proposals of the proposer (Cesarini et al., 2009; Smith and Silberberg, 2010). However, it has been shown that most proposers offered relatively fair proposals and responders chose to reject unfair allocations (Güth, 1995). The rate of the rejection of proposers increased, as the unfairness of the allocation proposal increases (Güth et al., 1982; Cooper and Dutcher, 2011; Lin et al., 2020). For example, when the share allocated to the recipient below 20%, the proposal was usually rejected (Camerer and Thaler, 1955).

Fairness considerations are a comparison of self-benefits with benefits of others and are a strong motivational driver in social interaction (Radke et al., 2012). An array of factors have been found to moderate the fairness consideration of people in gaming tasks such as the way of the game rules are presented, the relationship between the game parties, and the contextual information about the game (Kubota et al., 2013; Horat et al., 2016; Peterburs et al., 2017).

In social interactions, the intergroup relationship between the two sides of the game has a strong influence on the responses of people. When group members are determined, people tend to have positive preferences and attitudes toward their group (McAuliffe and Dunham, 2016). It was found that responders chose to accept unfair offers from ingroup proposers more often than unfair offers from outgroup proposers (McAuliffe and Dunham, 2016). Similar results have been found in other studies, where subjects were more likely to accept unfair offers when faced with in-group members (Wang et al., 2014, 2017). This pattern of ingroup bias is the basis for most forms of ingroup preferences (Wu and Zhou, 2013). In addition to the preferential treatment ingroup, people also expect the reciprocity of this preferential treatment (Gerard and Hoyt, 1974). Social identity theory assumed that people are encouraged to maintain a positive self-identity, including the social identity of the groups associated with them (Turner, 1975). They are, therefore, encouraged to positively evaluate members from their group. In addition, ingroup attachment and positive may make it easier for people to tolerate the selfishness of ingroup members relative to outgroup members (Brewer, 2007). However, there are studies that have found different results. In the second-party punishment of children, regardless of group membership, primary concern of children lay with fairness: Participants regularly offered equal splits and were more likely to reject unfair offers than fair offers (McAuliffe and Dunham, 2017). From the point of view of punishment, ingroup members would be punished more harshly than outgroup members for marginal fairness norm violations within the UG bargaining interactions (Mendoza et al., 2014). This suggests that the group member effect in fairness may be moderated by many factors such as age and environment and may require more research.

As one part of special education in colleges and universities, providing to deaf college students with a fair educational environment is an important pursuit of educational justice. Deaf college students have a unique culture, i.e., deaf culture, which also divides deaf people and people with normal hearing into two types of the social group. However, the social identity of deaf college students is conspicuous; some factors may affect the cultural identity of deaf college students. Like different language systems, the deaf students who grow up with sign language education tend to have an “immersive identity” or “bicultural identity” (Zhang, 2009) and are difficult to communicate with normal hearing college students (Stinson et al., 1996). This may make deaf college students have a tendency of deaf identity; great amount of studies have confirmed that deaf identity (Smiler and McKee, 2007; Leigh, 2010; Chapman and Dammeyer, 2017) and developed the theory of deaf identity (Glickman, 1996). This study aims to explore whether deaf college students are affected by group membership in the UG.

Focus on the sense of fairness of deaf college students in group membership would help to enrich such research and extend the scope of the study to special groups. In fact, we found little research on the perceptions of fairness of deaf college students when facing ingroup and outgroup members. In addition, inclusive education is a manifestation of equity in schools. The research on deaf college students has paid less attention to social functioning and if we fill this gap, it can better help us to understand deaf college students and promote the development of integrated education.

In this study, we recruited deaf college students to complete the UG with ingroup and outgroup membership. Meanwhile, their brain potentials were recorded. The feedback-related negativity (FRN) and P3 are two of the important electroencephalogram (EEG) components. The FRN is a negative deflection at frontocentral recording sites that peaks about 250 and 300 ms postonset of outcome feedback; source localization analysis has shown that the FRN is generated at the anterior cingulate cortex (ACC) (Gehring and Willoughby, 2002), a region that may reflect the conflict between cognition and emotion (Botvinick et al., 1999). It reflects an early reaction to a negative event such as an unfair offer (Yu et al., 2015).

Actually, Gehring and Willoughby (2002) called this negativity as the medial frontal negativity (MFN) first in their study, subsequent papers using gambling paradigms referred to the FRN or feedback negativity (Yeung et al., 2005). A good deal of studies has found the correlation between the FRN and loss/error by using correlation paradigm. Nonetheless, scholar has produced inconsistent interpretations of the FRN. A programmatic line of research indicating that this apparent negativity actually reflects the reward-related positivity (RewP) that is absent or suppressed following non-reward (Proudfit, 2015). In a gambling task, results found that breaking even elicited a relative negativity compared to gains; breaking even was just like losing (Holroyd et al., 2006). In another experiment, when breaking even could either be the best or worst possible outcome on a given trial, breaking even was always associated with a negativity (Kujawa et al., 2013). This reflected that losses and breaking even might be similarly categorized as unfavorable outcomes by the system that generates the negativity, even if breaking even is a relatively good result.

Another piece of evidence comes from N200, which has a striking resemblance to loss-related negativity in terms of timing, morphology, and scalp distribution (Holroyd, 2004). Moreover, N200 is generated whenever there is feedback, regardless of whether it contains information or not (Baker and Holroyd, 2009). Thus, the possibility arises that all the informative feedback in the gambling task triggers an N200 that is suppressed by the RewP in that time span.

By our review, does FRN represent a response to an unfavorable outcome or the RewP that is absent or suppressed following non-reward, variation in the FRN-reflecting activity related to negative feedback, positive feedback, or both is unclear (Holroyd, 2004); this deserves more investigation.

In studies exploring the effects of group membership on perceptions of fairness, it was found that the FRN was more negative for extremely and moderately unequal offers compared to equal offers in the ingroup interaction (Wang et al., 2017). This indicates that subjects were more surprised by the negative events given by the group members, eliciting the more negative FRN. This study focused on the fair decision-making performance of deaf college students in group membership and made the following hypothesis based on previous research: under unfair conditions, deaf college students would generate the more negative FRN facing ingroup members.

Another important EEG component is P3. P300 is a positive wave that peaks in the parietal-central region of the brain around 300–600 ms after the presentation of the outcome feedback and its amplitude is sensitive to the valence and magnitude of the outcome, with positive outcomes inducing a larger P300 than negative outcomes and the greater the reward, the greater the amplitude of P300 (Yeung and Sanfey, 2004). Research on the group bias on fairness consideration found that P300 amplitude in exposure to equal and advantageous inequal offers was more when they were opposed from their ingroup members (Keshvari et al., 2019). Ingroup members gave themselves greater benefits, which may have been consistent with group norms to the extent that subjects gained an advantage and induced greater P3. Based on previous research, this study proposes to hypothesize that under fair conditions, deaf college students will have greater P3 when faced with ingroup members compared to outgroup members.

The FRN and P300 are correlated, with P300 overlapping with the FRN in the time window and being equally sensitive to expectation violations (Hajcak et al., 2007). Based on this problem, we added principal component analysis (PCA) to the windowed difference wave approach to separate the FRN and P300 (Foti et al., 2011).

Due to the lack of attention to deaf college students, exploring the fairness consideration of deaf college students not only helps to understand their social interaction, but also provides further support for the integration of higher education.

## Materials and Methods

### Participants

A total of 29 deaf college students, including 13 males and 16 females, randomly selected from Chongqing Normal University, aged between 18 and 24 years (M = 21.34 years, SD = 1.42 years), were participated in this study. They were paid 30 Chinese Yuan (about $4.5) as basic payment and were informed that additional monetary rewards would be paid according to the offers of proposers and their decisions in the task.

They were all sign language users with normal or corrected-to-normal vision and had no history of neurological disorders or mental disease. A written informed consent was obtained from all the subjects prior to the ERP experiment. All the subjects were included in the final data analysis. This experiment was approved by the Local Ethics Committee of Chongqing Normal University.

### Experimental Design and Stimulation

A 2 (group membership: ingroup vs. outgroup) × 3 (proposal type: fair/moderately unfair/extremely unfair) within-subjects design was created. When the proposer was deaf college students named ingroup, the normal hearing college students (experimental assistants in this study) were outgroup. Fair offers could be 5 Yuan (out of 10 Yuan), moderately unfair offers could be 3 or 4 Yuan, and extremely unfair offers could be 1 or 2 Yuan.

Before the experiment, the photos of two experimental assistants were collected and presented in the program. The experimental stimulus is gray Chinese characters or numbers on the black screen. Digital font Courier, size 36. Allocate font for proposal and final result display, font size 36. The pictures of proposers are grayscale pictures with 300 × 300 pixels.

### Procedures

The experimental procedure was prepared using E-prime 3.0. In the UG, subjects are responders, two are experiment assistants, one is ingroup proposer and the other is outgroup proposer. Prior to participation, all the subjects were told the experimental procedure and signed informed consent form. Participants were told that they will play a money sharing game, totally 10 dollars each time, with the proposer making the offer and the responders choosing to accept or reject the offer. If responders accept the offer, both the sides get the amount offered, but if responders reject, both the sides get zero Yuan. The fair offer is 5/5, the moderately unfair offers are 6/4 and 7/3, and the extremely unfair offers are 8/2 and 9/1. Before the slash is the portion that the proposer divides between himself and after the slash is what the responder gets.

**FIGURE 1:**
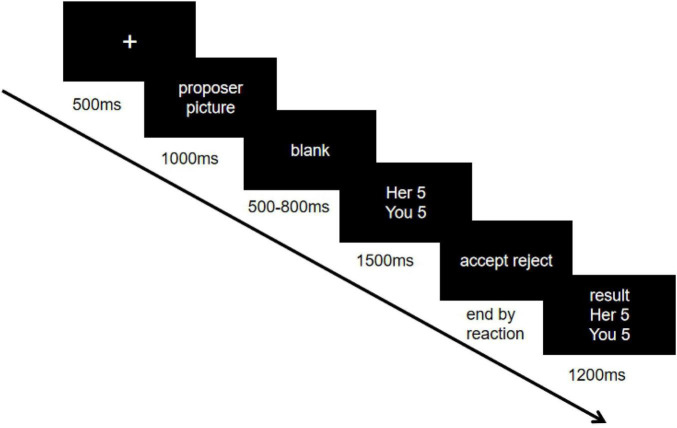
An example of the ultimatum game (UG). First, a cross will appear in the center of the screen for 500 ms, after that the picture of the gaming object will appear on the screen for 1,000 ms, after a random empty screen from 800 to 1,500 ms the allocation scheme proposed by the proposer will be presented for 1,500 ms, after that the responder will see the interface of the response and choose to accept or reject it to enter the next process, and finally the result of this round will be presented for 1,200 ms.

The participants completed the EEG experiment and then sat in a quiet room and began reading the instructions, which were explained to the deaf college student by a professional sign language interpreter. It was explained to the subject that final result would determine his or her reward. To enhance the realism of the experimental situation, there were two assistants, a normal hearing college student (outgroup members) and a deaf college student (ingroup members). They were arranged to play with the subject, explaining the experimental procedure to both the sides, and having the proposer pretend to play with the subject in another laboratory, where the procedure was actually designed. Before the formal experimental task, each allocation scheme was presented twice for the subjects to practice. The experiment consisted of 360 trials, 60 for each condition and 180 for each of the ingroup and outgroup conditions. Figure 1 shows an example of all the trials.

### Electroencephalogram Recording Data Reduction and Analysis

The EEG was recorded from 64 electrode locations arranged in the standard 10–20 layout using Brain Vision Recorder software. During recording, the EEG data were referenced to the average voltage across channels, sampled at 1,000 Hz, and amplified (Brain Vision LLC, Morrisville, NC, United States) and filtered through a passband of DC ∼280 Hz. Impedances were below 5 kΩ.

The EEG data were preprocessed offline and analyzed using Matrix&Laboratory (MATLAB) R2016a (MathWorks, Natick, MA, United States) and EEGLAB 13.6.5b components. The analysis was performed using the mean value of both the papillae as a reference, with a filtered bandpass of 0.1–20 Hz. Artifacts of ±80 μV at all the electrodes were also excluded. The analyses time interval was from 200 ms before to 1,000 ms after the presentation of the proposal type. Took the first 200 ms of the proposal type as the baseline. Trials with severe electromyogram (EMG) interference were excluded and eye movement artifacts were corrected by independent component analysis (ICA) algorithm.

Based on visual observations of the grand average waveforms and the previous ERP studies (Alexopoulos et al., 2012; Wu and Zhou, 2013), we averaged the ERP amplitude from the time range 110–150, 200–250, 300–350, and 400–600 ms postoffer presentation for the N1, P2, FRN, and P3 analyses, respectively. According to the scalp distribution and previous reports, we selected nine electrode sites (Fz, F1, F2, FCz, FC1, FC2, Cz, C1, and C2), six electrode sites (FCz, FC1, FC2, Cz, C1, and C2), and seven electrode sites (Fz, F1, F2, FCz, FC1, FC2, and Cz) in the frontal and central areas for the N1, P2, and the FRN analysis and nine electrode sites (Pz, P1, P2, POz, PO3, PO4, Oz, O1, and O2) in the central-parietal areas for P3 analysis.

After we averaged the ERP data for the six conditions, apply temporospatial PCA to distinguishing the FRN from overlapping responses. PCA is a factor analytic approach that can be used to parse the observed ERP waveform into underlying constituent components (Dien, 2010). This analysis was conducted using the latest ERP PCA Toolkit version developed by Dien (2010).^1^ A temporal PCA was first performed on the data to capture variance across time points. This PCA used all the time points from the averaged ERP of each participant as variables and it considered participants, trial types, and recording sites as observations. Promax rotation was used and nine temporal factors were extracted based on the resulting Scree plot (Cattell, 1966). For each temporal factor, this analysis yielded factor scores for each combination of electrode, participant, and trial type, representing the amount of activity in the original data captured by that factor. The spatial distribution of these factor scores was then analyzed using spatial PCA. This PCA used all the recording sites as variables and it considered all the participants, trial types, and temporal factor scores as observations. A separate spatial PCA was performed for each of the nine temporal factors. Infomax rotation was used and based on the averaged Scree plot for all the nine temporal factors, four spatial factors were extracted, yielding 36 unique factor combinations. The covariance matrix and Kaiser normalization were used for each PCA. The waveforms for each factor were reconstructed (i.e., converted to microvolts) by multiplying the factor pattern matrix with the SDs.

The data were statistically analyzed using the IBM SPSS Statistics version 25.0 (SPSS Incorporation, Chicago, IL, United States). The acceptance rate of the fair decision-making of subjects was collected for behavioral data and the mean wave amplitude of each component was selected for the EEG data. A two-factor repeated measures ANOVA of 2 (group: ingroup vs. outgroup) × 3 (proposal type: fair/moderately unfair/extremely unfair) were conducted for acceptance rate and mean wave amplitude, respectively.

## Results

### Behavior Results

The acceptance rate of different group relationships is shown in Table 1.

**TABLE 1:** Acceptance rate of subjects under different group membership (M ± SD) unit:%.

	Fairness	Moderately unfair	Extremly unfair
Ingroup	0.99 ± 0.05	0.54 ± 0.30	0.09 ± 0.17
Outgroup	0.98 ± 0.55	0.56 ± 0.21	0.08 ± 0.11

The main effect of the proposal type of acceptance rate was significant, *F*(2,27) = 340.81, *P* < 0.001, η*^2^* = 0.96. The simple effect showed that the acceptance rate of fair proposal (0.98 ± 0.01%) was significantly higher than that of moderate unfair proposal (0.54 ± 0.06%) and extremely unfair proposals (0.08 ± 0.03%), with moderate unfair proposals being significantly larger than extremely unfair proposals. The main effect of group relationship was not significant and the interaction between group and proposal type was not significant.

### Electroencephalogram Results

**FIGURE 2:**
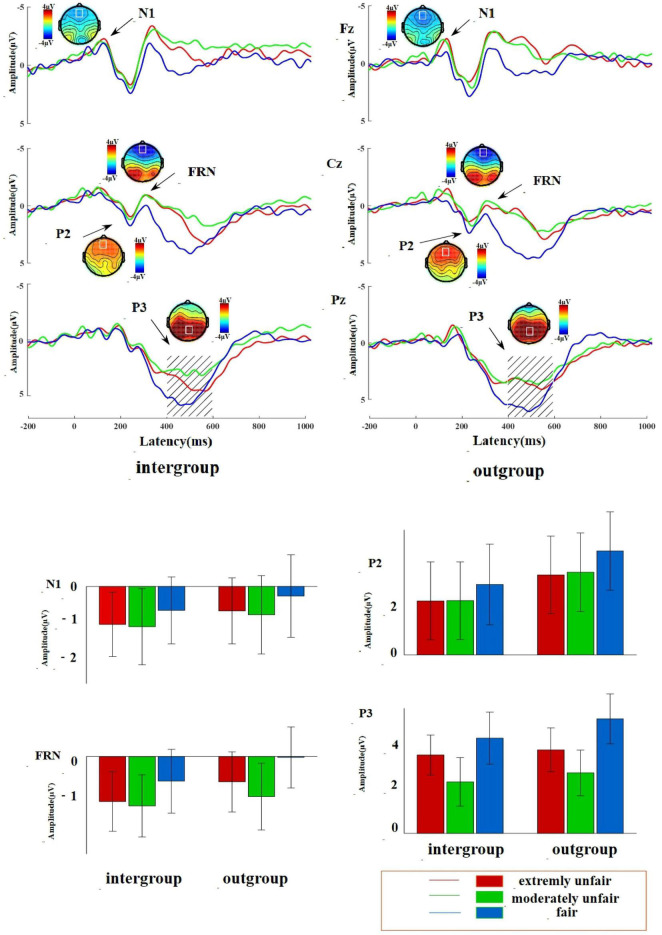
Topographic waveforms and histograms of different group relationships, with topographic waveforms at the upper part and histograms at the lower part. The above picture shows the electroencephalogram (EEG), topographic map, and bar chart of deaf college students and healthy listening college students under different proposal types; the upper part of black solid line is divided into topographic map and waveform map and the lower part is divided into bar chart; the red solid lines and bars represent extremely unfair conditions, the green solid lines and bars represent moderate unfair conditions, and the blue solid lines and bars represent fair conditions.

See Table 2 for variance analysis of the EEG data with different group membership and see Figure 2 for the EEG topographic waveform.

**TABLE 2:** The ANOVA of the electroencephalogram (EEG) data in different group memberships.

	Group membership			Proposal type			Group membership × proposal type		
	*F*	*P*	η^2^	*F*	*P*	η^2^	*F*	*P*	η^2^
N1	3.20	0.09	0.10	3.40	0.04	0.20	0.02	0.98	0.00
P2	9.56	0.00	0.26	1.77	0.19	0.12	0.31	0.74	0.02
FRN	4.55	0.04	0.14	3.44	0.04	0.20	0.38	0.69	0.03
P3	3.67	0.06	0.12	8.38	0.00	0.38	0.97	0.40	0.07

### N1

The ANOVA analysis for the N1 identified that the main effect of group membership was not significant, *F*(1,28) = 3.20, *P* > 0.05, η^2^ = 0.10. The main effect of proposal type was significant and the average amplitude induced by moderate unfair proposal (–1.16 ± 0.62 μV) was significantly larger than fair proposal (–0.57 ± 0.59 μV), *F*(2,27) = 3.40, *P* < 0.05, η*^2^* = 0.20.

### P2

The main effect of group membership was significant; the average amplitude induced by ingroup members (1.45 ± 0.95 μV) was significantly lower than that of outgroup members (2.25 ± 0.95 μV), *F*(1,28) = 9.56, *P* > 0.05, η*^2^* = 0.10. The interaction between group membership and proposal type was not significant, *F*(2, 27) = 0.31, *P* = 0.74, η*^2^* = 0.02. However, this article focuses on the simple effects of group membership under different proposal types and simple effects can be significant when interactions are not significant (Umesh et al., 1996; Tybout et al., 2001). When the purpose of this study is to focus only on the main simple effects, it is not a prerequisite that the interactions are significant and the results for simple effects are plausible at this point (Hayes, 2005, p. 447). Simple effects found that under the fair offer condition, deaf college students induced greater P2 when faced with ingroup members (2.66 ± 1.00) compared to outgroup members (1.80 ± 1.03) (*P* = 0.035).

### Feedback-Related Negativity

The main effect of group membership was significant, *F*(1,28) = 4.55, *P* < 0.05, η*^2^* = 0.14; when the offer was assigned to ingroup members, the induced average amplitude (–1.33 ± 0.99 μV) was significantly larger than that of outgroup members (–0.74 ± 1.02 μV). The main effect of proposal type was significant and the average amplitude induced by moderate fair proposal (–1.50 ± 1.06 μV) and extremely unfair proposal (–1.18 ± 0.98 μV) was significantly larger than fair proposal (–0.43 ± 1.03 μV), *F*(2,27) = 3.44, *P* < 0.01, η*^2^* = 0.20.

### P3

The ANOVA analysis for P3 identified that the main effect of group membership was not significant, *F*(1,28) = 3.67, *P* > 0.05, η*^2^* = 0.12. When the distribution scheme was proposed by ingroup members (3.83 ± 1.13 μV), the induced average amplitude was significantly smaller than that of outgroup members (4.41 ± 1.15 μV). The main effect of proposal type was significant and the average amplitude induced by fair proposal (5.36 ± 1.29 μV) was significantly larger than that induced by moderate unfairness (2.85 ± 1.19 μV) and extremely unfair proposal (4.14 ± 1.05 μV), while the average amplitude induced by extremely unfair proposal was significantly larger than that induced by moderate unfairness, *F*(2,27) = 8.38, *P* < 0.01, η^2^ = 0.38. The interaction for P3 is similarly insignificant, *F*(2,27) = 0.97, *P* = 0.39, η^2^ = 0.067, but as with P2, we focus only on the simple effects of group membership in the proposed type condition. Simple effects found that under the fair offer condition, deaf college students induced greater P3 when faced with ingroup members (5.86 ± 1.23) compared to outgroup members (4.87 ± 1.33) (*P* = 0.015).

### Principal Component Analysis Results

Of the 36 total factor combinations yielded by PCA, 11 total factor combinations accounted for at least 1% of the total variance in the data. Based on our above results, we chose four factors corresponding to N1, P2, FRN, and P3 (Table 3). The EEG components that were not considered either had no clear time window because their contribution was too low or did not correspond to the known ERP components. An ANOVA on the four components did not find an interaction between group membership and proposal type.

**TABLE 3:** Principal component analysis (PCA) factor combinations selected for statistical analysis.

Corresponding ERP component	Temporospatial factor combination	Unique variance explained (%)	Temporal loading peak (ms)	Spatial distribution
N1	TF5/SF1	1.1	132	Frontocentral negativity
P2	TF4/SF1	2.5	220	Frontal positivity
FRN	TF8/SF1	1.1	388	Frontocentral negativity
P3	TF1/SF1	9.8	456	Parietal positivity

**FIGURE 3:**
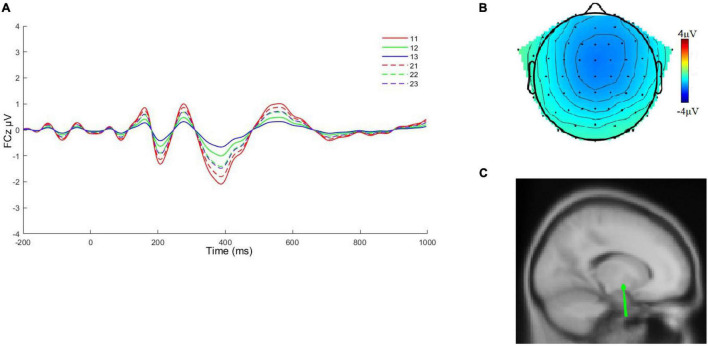
**(A)** The average waveform of the feedback negativity at FCz for six conditions. 11: ingroup, extremely unfair; 12: ingroup, moderately unfair; 13: ingroup, fair; 21: outgroup, extremely unfair; 22: outgroup, moderately unfair; 23: outgroup, fair. **(B)** Topography of deaf college students in the face of extremely unfair conditions. **(C)** The dipole source associated with time factor 8/spatial factor 1 (TF8/SF1).

Figure 3 shows the waveforms and topographies associated with time factor 8/spatial factor 1 (TF8/SF1) and PCA factor corresponding to the FRN and head topography of deaf college students under extremely unfair condition. Wave peaks are generated at frontal-central zero (FCz). Faced with an extremely unfair proposal, the deaf college students produced the most negative component at a time window of 388 ms. Source localization of this condition identified the putamen as a likely neural generator, with Talairach coordinates of (8, –4, –8) and residual variance (RV) of 3.6%.

**FIGURE 4:**
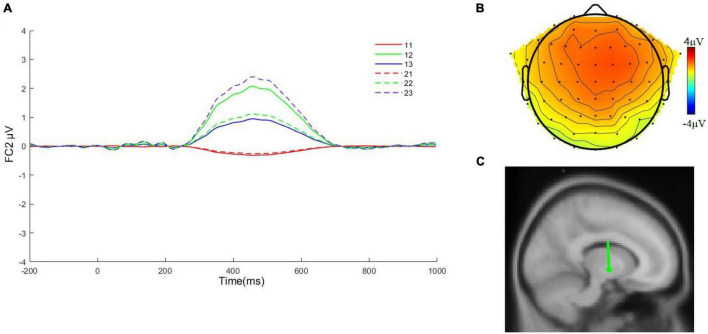
**(A)** The average waveform of P3 at FC2 for six conditions. 11: ingroup, extremely unfair; 12: ingroup, moderately unfair; 13: ingroup, fair; 21: outgroup, extremely unfair; 22: outgroup, moderately unfair; 23: outgroup, fair. **(B)** Topography of deaf college students in the face of fair conditions. **(C)** The dipole source associated with time factor 1/spatial factor 1 (TF1/SF1).

Figure 4 shows the waveforms and topographies associated with time factor 1/spatial factor 1 (TF1/SF1) and PCA factor corresponding to P3. Although we did not find an interaction between group membership and offer type under PCA, interesting results can be seen based on the waveform plots, where normal college students produced the largest waveform when faced with a fair offer, while deaf college students produced the largest waveform when faced with a moderately unfair offer, indicating that the fair offer here was not the most favorably valenced for deaf college students. The results of coupled polariton localization suggest that the origin of P3 component may be in the middle of the cingulate gyrus, with Talairach coordinates of (–8, –7, –3) and RV of 5.3%.

## Discussion

According to the neural compensation effect, the results showed that group membership can moderate the fair decision-making of deaf college students and they tend to interact with ingroup members. We found that the behavioral results indicated that the more unfair the proposal type, the lower the acceptance rate, but the group membership has no significant difference in acceptance rate. The EEG results found that N1 main effect margin is significant and the average amplitude of ingroup members was greater than outgroup members. The main effect of P2 is significant and the average amplitude of ingroup members was smaller than outgroup members. The main effect of the FRN is significant and the average amplitude of ingroup members was greater than outgroup members. The main effect margin of P3 is significant and the average amplitude of ingroup members was smaller than outgroup members.

First, the behavioral results reflected the rejection of unfairness. The more unfair offer type, the lower the acceptance rate of the subjects and the moderately unfair offers had an acceptance rate of about 50%. We did not find any differences in acceptance rates among deaf college students when faced with different group memberships. This is consistent with the previous study such as decision-making behavior in the UG task was manipulated by the perceptions of fairness of subjects rather than their assessments of the proposer (Mendoza et al., 2014). It is possible that the absence of explicit information about the grouping of subjects led to a no effect; we found an effect of grouping on the EEG, which is inconsistent with the behavioral outcome probably because the behavior and the EEG differ in time course; the study also found that group bias was induced to children aged 6–9 years, but children rejected unfair offers from both the ingroup and outgroup members and there was no significant difference (Gonzalez et al., 2020). This suggests that deaf college students may have the same decision-making patterns as children and that equity norms outweigh group bias in their behavior.

Second, the EEG results found a main effect of N1, with subjects evoking significantly larger mean wave amplitudes when confronted with offers from ingroup members than from outgroup members, a result consistent with the previous EEG studies (Kubota and Ito, 2007; Boudreau et al., 2009; Ito and Bartholow, 2009). This may be due to a combination of group norms and expectations of subjects; in terms of group norms, when dealing with ingroup members, people generally adopt the principle of reciprocity and when ingroup members violate norms, they receive greater punishment than outgroup members (McAuliffe and Dunham, 2016); in terms of expectations of subjects, when dealing with deaf college students who are also ingroup members, subjects expect a fairer offer from the proposer and when the proposer made an unfair offer, it violated the expectation of the subject and produced a stronger response, whereas the subject did not have this expectation for outgroup members (Balliet et al., 2014).

Third, we found that under fair conditions, deaf college students induced greater P2 when faced with ingroup members compared to outgroup members. P2 is a positive component that appears around 200 ms after stimulus presentation and is often found in cognitive tasks involving workload and attentional processes (Horat et al., 2016). Our results suggested that subjects allocated more attentional resources when gaming with ingroup members. In addition, some researchers have argued that interest congruence has stronger motivational and perceptual salience than conflict of interest conditions (Boudreau et al., 2009). The results may indicate that subjects who were confronted with ingroup members were more concerned with the distribution of benefits and with ingroup members. Therefore, P2 induced by ingroup members is bigger.

Then, a main effect of the FRN group membership was found, with subjects inducing significantly larger mean wave amplitudes than outgroup members when ingroup members assigned proposals. The FRN component represents fair supervision, an event-related potential (ERP) thought to originate in the dorsal anterior cingulate cortex (dACC), with both the loss outcomes and deviations from expectations triggering the more negative FRNs and the significance of the group relationship suggests that ingroup members offers deviated more from the expectations of subjects. However, inconsistent with previous research, we did not find that under the unfair proposal, deaf college students induced the more negative FRN when faced with ingroup members than with outgroup members (Wang et al., 2016, 2017). This may be due to the following reasons: the group manipulation of subjects was not yet strong enough, the results of N1 also did not interact, both the N1 and FRN responded to negative results, and it is possible that deaf college students are more sensitive to positive ingroup bias.

Lastly, the results for P3 found a significant main effect margin for the group, with the average wave amplitude induced by subjects when the offer was made to an outgroup member, i.e., a hearing college student, slightly larger than that of ingroup members. Under fair conditions, deaf college students induced greater P3 when faced with ingroup members compared to outgroup members. In the UG, P3 played an important role in the valuation of events and was more sensitive to rewards and subjects rated the proposal of ingroup members higher than outgroup members indicating that deaf college students were more concerned about ingroup equity. In addition, P3 was associated with higher cognitive operations and motivational awareness and the stronger P3 generated by subjects when playing with ingroup hearing college students may indicated that deaf college students generated stronger benefit motivation, indicating that deaf college students were more concerned about ingroup fairness.

We successfully separated the FRN and P3 using PCA, with the FRN peaking at the FCz electrode position and P3 peaking at FC2. However, we found that the FRN peaks at the 388 ms time window, which is slightly outside the time window range of previous studies in the literature. However, in conjunction with our ultimatum task, we still consider this to be relatively normal, the reasons for this result remain to be examined.

For the FRN, we did not find an interaction between group membership and proposal type, either in the traditional time window analysis method or in PCA. However, deaf college students produced the more negative FRNs when confronted with group membership. This study showed that the FRNs were sensitive to the valence of the outcome, but not to the size of the outcome and that the FRN distinguished between monetary gains and losses, but was comparable to larger losses compared to smaller losses (Sato et al., 2005). In our experiments, there are only fair and unfavorable unfair conditions and deaf college students do not receive favorable unfair assignments from group members. After several interactions, it is possible that the fair condition may also be perceived as less favorable, as shown in the results of P3. From this aspect, the FRN is a feedback on the perceived unfavorable results; this is similar to the views of other researchers (Hajcak et al., 2007).

Indeed, that is say, the FRN tracks the relative valence of outcomes in a direct context, making the magnitude of the FRN elicited by neutral feedback dependent on whether the alternative wins or loses on that trial (Holroyd et al., 2004, 2006). This means that the FRN is related to personal expectation and the size of personal expectation is not the same and for individuals, expectation is only good or bad without size. Together, these lines of research indicate that the FRN reflects a process in which outcomes are evaluated as either better or worse than expected.

It is unclear to researchers whether changes in the FRN responses are related to negative feedback, positive feedback, or both; previously, the FRN was typically interpreted as a negative ERP component that was augmented for feedback indicative of error and irreversibility, presumably reflecting a neural process that tracks the occurrence of adverse outcomes. Instead, the FRN is quantified as the difference in values between negative and positive feedback (Foti and Hajcak, 2009). Although this approach isolates changes in ERPs associated with the feedback valence, it cannot attribute such changes to a specific outcome. It has been suggested that the FRN amplitude itself is not meaningful and that the apparent decrease in the amplitude of one component may instead be due to the appearance of an overlapping component with opposite polarity (Luck, 2005).

This component is P3. In fact, just as our experiments need to discover simple effects under the influence factor, we need to separate the two related components to know what role they each play.

Taken together, previous research suggests that the effect of group membership on fairness considerations is ambiguous at the behavioral outcome. Our results found that neither ingroup nor outgroup membership influenced fairness decisions among deaf college students, contrary to previous research (Wang et al., 2017). However, the results of this study on the groups of children were consistent with this study (Gonzalez et al., 2020). This may indicate that there are other influences between group membership affecting equity considerations such as age of the group, manipulated groups, or naturally forming groups. On the EEG results, we found a very significant group bias effect, i.e., under fair conditions, deaf college students induced more positive P2 and P3 when faced with ingroup members compared to outgroup members. Many studies have found enhanced P3 when subjects are faced with fair offers (Wu and Zhou, 2009; Wu et al., 2011; Yu et al., 2015). Moreover, results consistent with this study were found in some special population subjects. In a study of Chinese and foreign children, it was found that allocations involving outgroup children also elicited diminished P300 amplitudes and enhanced delta responses when subjects faced conflicts between equality and efficiency, rather than allocations within ingroup children (Yu et al., 2021). Another research investigated the behavioral and ERP responses of healthy people playing the UG game with Down syndrome (DS) and typical development (TD) proposers found that a higher P300 amplitude was detected when participants faced fair offers from TD compared to DS fair offers. These evidences suggest that ingroup bias influences the neural processes of equity consideration.

In summary, we found that ingroup bias is also present among deaf college students, which has positive implications for understanding this group and promoting inclusive education.

## Conclusion

In this study, we found that group membership moderated the fair consideration of deaf college students in the UG task across different time courses. In conclusion, this study found that deaf college students had an ingroup bias in the UG and paid more attention to fairness ingroup, reflecting in the two EEG components, P2 and P3. This provides some evidence for the fairness characteristics of special populations.

### Limits

This study may have the following limitations. First, the sample size of subjects is not enough and the small number of deaf college students is one of the reasons; moreover, the problem of funds and time. Second, deaf college students were not classified in more detail such as the level of hearing impairment. In future studies, medical diagnosis of hearing impairment can be used to further distinguish the effects of different hearing levels.

## Data Availability Statement

The original contributions presented in the study are included in the article/supplementary material, further inquiries can be directed to the corresponding authors.

## Ethics Statement

This experiment was approved by the Local Ethics Committee of Chongqing Normal University. The patients/participants provided their written informed consent to participate in this study.

## Author Contributions

YG and XD contributed to the concept and design of the study. YG and LY contributed to data acquisition, analysis, and interpretation. XC contributed to subjects’ recruitment and manuscript revision. QX provided technical support. YG, JJ, and XD were responsible for the article, guided the experiment, and modified the manuscript to ensure the integrity and accuracy of every part of the work. All authors approved the final version to be published.

## Conflict of Interest

The authors declare that the research was conducted in the absence of any commercial or financial relationships that could be construed as a potential conflict of interest.

## Publisher’s Note

All claims expressed in this article are solely those of the authors and do not necessarily represent those of their affiliated organizations, or those of the publisher, the editors and the reviewers. Any product that may be evaluated in this article, or claim that may be made by its manufacturer, is not guaranteed or endorsed by the publisher.

## Footnotes

1
http://sourceforge.net/projects/erppcatoolkit/
